# Metacognitive Accuracy Improves With the Perceptual Learning of a Low- but Not High-Level Face Property

**DOI:** 10.3389/fpsyg.2019.01712

**Published:** 2019-07-24

**Authors:** Benjamin Chen, Matthew Mundy, Naotsugu Tsuchiya

**Affiliations:** ^1^School of Psychological Sciences, Faculty of Biomedical and Psychological Sciences, Monash University, Melbourne, VIC, Australia; ^2^Monash Institute of Cognitive and Clinical Neuroscience, Monash University, Melbourne, VIC, Australia

**Keywords:** conscious and unconscious memory, face perception and cognition, metacognition, perceptual learning, memory and learning

## Abstract

Experience with visual stimuli can improve their perceptual performance, a phenomenon termed visual perceptual learning (VPL). VPL has been found to improve metacognitive measures, suggesting increased conscious accessibility to the knowledge supporting perceptual decision-making. However, such studies have largely failed to control objective task accuracy, which typically correlates with metacognition. Here, using a staircase method to control this confound, we investigated whether VPL improves the metacognitive accuracy of perceptual decision-making. Across 3 days, subjects were trained to discriminate faces based on their high-level identity or low-level contrast. Holding objective accuracy constant across training days, perceptual thresholds decreased in both tasks, demonstrating VPL in our protocol. However, whilemetacognitive accuracy was not affected by face contrast VPL, it was decreased by face identity VPL. Our findings couldbe parsimoniously explained by a dual-stage signal detection theory-based model involving an initial perceptual decision-making stage and a second confidence judgment stage. Within this model, internal noise reductions for both stages accounts for our face contrast VPL result, while only first stage noise reductions accounts for our face identity VPL result. In summary, we found evidence suggesting that conscious knowledge accessibility was improved by the VPL of face contrast but not face identity.

## Introduction

The relationship between conscious perception and learning remains a central topic in cognitive neuroscience (Bayne et al., 2009). This topic has been investigated across various conscious perception and learning paradigms. For instance, within the visual perceptual learning (VPL) paradigm, the degree to which subjects improve perceptual performance in a visual task is dependent on their experience with the stimuli used in the task (Fahle and Poggio, 2002). In relation to conscious perception, one line of VPL research has investigated whether conscious experience of visual stimuli is necessary for their VPL, by employing consciously invisible stimuli as the target of VPL (Watanabe et al., 2001; Seitz et al., 2009). In contrast, another line of research has investigated whether VPL improves conscious accessibility to the learned information supporting its perceptual decision-making. Addressing the latter, recent studies have found that the VPL of simple object properties (e.g., shape) improved subjects’ confidence in their perceptual decisions (Schwiedrzik et al., 2011; Bertels et al., 2012; Schlagbauer et al., 2012). However, because VPL increased objective task accuracy in these studies, it remains possible that the improvements in confidence judgements were a consequence of increased objective accuracy (Galvin et al., 2003; Maniscalco and Lau, 2012; Barrett et al., 2013), rather than improved conscious accessibility to the knowledge supporting perceptual decision-making (see however, Schwiedrzik et al., 2011).

Here, to address this potential confound, we use an adaptive staircase procedure to hold objective task accuracy constant (e.g., 75% correct). Using such procedures, VPL is quantified as a decrease in the stimulus intensity needed to maintain a fixed level of task accuracy (Gold et al., 2010). Within this framework, the critical question now becomes whether or not VPL improves subjects’ ability to consciously access learned perceptual information. Assuming that a high confidence rating that accompanies with a correct decision reflects high degree of conscious accessibility of information for the decision, we quantify conscious accessibility as metacognitive accuracy in a Type-II signal detection task. In a Type-II task, subjects discriminate their correct from incorrect responses using confidence ratings (Galvin et al., 2003).

**FIGURE 1:**
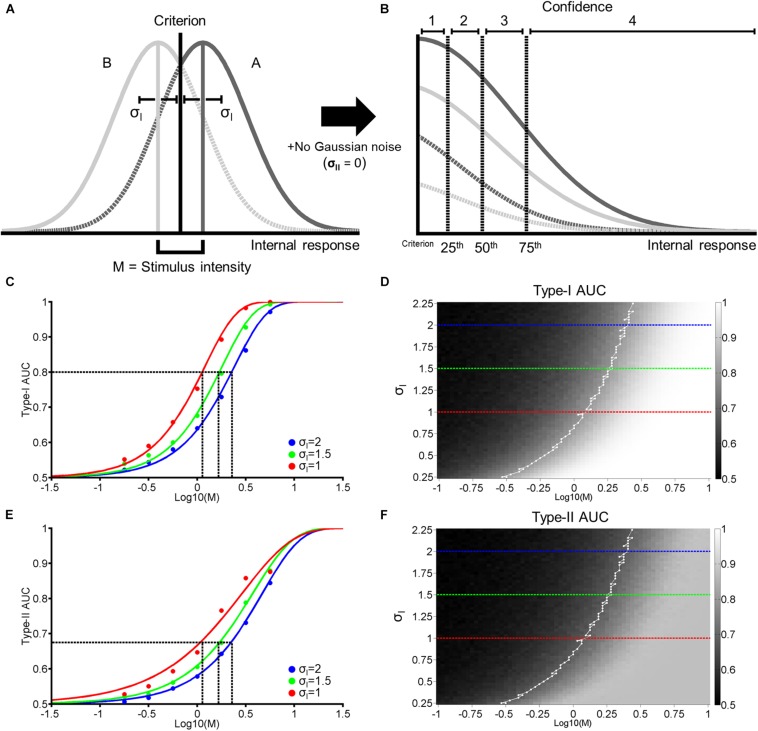
Single-stage signal detection model. **(A)** Internal response probability densities for face A (solid and dashed dark gray) and B (solid and dashed light gray) alternatives. We assume both distributions are Gaussian with equal variance (σ_*I*_). When A and B differ physically in some magnitude in terms of morph distance in Exp. 1 and contrast difference in Exp. 2, we assume that the distance between the mean of each distribution is M. We further assume an unbiased perceptual response criterion with a value of zero (black vertical line). From this criterion, we classify perceptual responses as hits (solid dark gray), misses (dashed dark gray), correct rejections (solid light gray), or false alarms (dashed light gray). **(B)** To obtain confidence rating for each trial, we first took the absolute value of internal responses. Then, we assigned confidence ratings (1–4) according to set percentiles (dashed vertical lines; from left to right: 25th, 50th, and 75th percentiles) from the perceptual response criterion across 10,000 trials. **(C)** Hypothetical psychometric functions corresponding to Type-I Area Under the Curve (AUC) with different levels of noise (Blue: σ_*I*_ = 2, green: σ_*I*_ = 1.5, and red: σ_*I*_ = 1). Vertical dashed lines indicate the thresholds at each level of noise to attain Type-I AUC of 0.8 (horizontal dashed line). **(D)** Surface plot for Type-I AUC as a function of M and σ_*I*_, with dashed blue, green, and red lines corresponding to the σ_*I*_ parameters in **(C)**. The white line in **(D)** represents the M and σ_*I*_ parameters corresponding to Type-I AUC around 0.8 (0.79–0.81). **(E,F)** correspond to Type-II AUC with the same format as **(C,D)**, respectively.

In this paper, we first present a simple model which simulates a single stage for both perceptual decision-making and confidence judgements. In this model, we relate stimulus difference, objective task accuracy (or Type-I performance), and metacognitive accuracy (or Type-II performance; Figure 1). To test the prediction of this model, we performed two experiments where we trained subjects in the VPL of face identity (Experiment 1) or face contrast (Experiment 2). We found that the prediction of the single-stage model was consistent with the results in Exp. 2., but not those in Exp. 1. To resolve this inconsistency, we propose an alternative model, which simulates two separate stages, one for perceptual decision-making and the other for confidence judgements, which can account for the results in both Exp. 1 and 2.

## Model I: Single-Stage Noise Reduction Model

Using a framework based on signal detection theory (SDT; Macmillan and Creelman, 2005), we propose a generative model that relates stimulus difference and Type-I and II performance (Figure 1). Firstly, we construct two Gaussian probability density functions. The functions depend on the strength of stimulus difference (M), which generates a distribution of hypothesized internal responses (e.g., neural responses in the brain) for two response alternatives. In our experimental task, these correspond to face A and B alternatives (see Exp. 1 and 2 Method). We define the difference in the means of both distributions as M, with the mean of the face A and B distributions located at +M/2 and –M/2 from 0, respectively. We assume that as the physical difference between the two stimulus alternatives increases, the greater the difference of the internal responses becomes. Furthermore, we assume that the two distributions have the same standard deviation (σ_*I*_), and that subjects adopt an unbiased and optimal perceptual decision criterion (i.e., a decision criterion value of zero; Figure 1A).

Based on the value of M and σ_*I*_ (the 2 free parameters of this model), we generate around 5000 random values for each response alternative (Figure 1A, Gaussian distributions). For each random value, we assign a perceptual response (i.e., a response of A if a random value is more than the decision criterion, and a response of B otherwise). We then assign a confidence rating from 1 to 4 to each perceptual response according to the distance between a random value to the decision criterion. Specifically, we took the absolute value of random values from both A and B response distributions and calculated the 25^*th*^, 50^*th*^, and 75^*th*^ percentiles of the cumulated distributions from the decision criterion. We then accordingly assigned confidence ratings (1–4) in equal proportion (25% each) to random values based on these quantiles (Figure 1B). Thus, in this model, we assume that perceptual decision-making and confidence judgements are based on the same signal (Galvin et al., 2003; Kepecs et al., 2008; Maniscalco and Lau, 2012; Barrett et al., 2013; Sanders et al., 2016). We term this model the ‘single-stage model’.

Based on perceptual responses and confidence ratings over 10,000 random values, we next compute objective accuracy as the Area Under the Type-I receiver operating characteristic (ROC) Curve, and metacognitive accuracy as Type-II Area Under the ROC Curve (AUC; see Exp. 1 Method). Figure 1C demonstrates the relationship between Type-I AUC (y-axis) and stimulus difference, M (x-axis), at 3 different levels of noise (blue, green, and red for σ_*I*_ = 2, 1.5, and 1, respectively). Figure 1D encodes Type-I AUC in color-scale (black for 0.5 and white for 1) as a function of M (x-axis) and σ_*I*_ (y-axis). Psychometric functions in Figure 1C correspond to the height of the surface plot in Figure 1D at respective levels of σ_*I*_, indicated by dashed horizontal lines (blue, green, and red for σ_*I*_ = 2, 1.5, and 1, respectively).

Figure 1E demonstrates the relationship between Type-II AUC and M at the same 3 noise levels as Figures 1C,F encodes Type-II AUC in color-scale as a function of M and σ_*I*_.

This model clarifies the relationship between stimulus difference (M) and Type-I and II AUC, and demonstrates how they are modulated by the internal noise associated with perceptual decision-making (σ_*I*_). Firstly, both Type-I and II AUC monotonically increase as a function of M or σ_*I*_, when the other variable is held constant (Figures 1D,F). Indeed, the correlation between Type-I and II AUC over the 4225 data points (65 levels of M × 65 levels of σ_*I*_) shown in Figures 1D,F was near perfect, with a correlation coefficient of 0.99. From this strong correlation, we can derive a simple prediction about how VPL would affect both Type-I and II AUC. Consider the hypothesis that VPL decreases the internal noise (i.e., σ_*I*_) associated with perceptual decision-making (Dosher and Lu, 1998). Under this hypothesis, if we maintain Type-I AUC at 0.8 using a staircase method (as we do in the main experiments), then we should observe that stimulus difference (M) thresholds should decrease with VPL (dashed vertical lines in Figure 1C). Furthermore, we should also observe that Type-II AUC will stay constant (i.e., around 0.68) with VPL at the corresponding M values (dashed vertical lines in Figure 1E), due to the strong correlation between Type-I and II AUC. In the following two experiments, we will test this prediction: whether Type-II AUC remains constant as subjects are trained to discriminate face identity or face contrast in a VPL task.

### Experiment 1: Face Identity VPL

#### Method

##### Subjects

Twenty subjects (13 female and 7 male, *M*_*age*_ = 24.5, *SD* = 5.47) were recruited from Monash University and monetarily reimbursed for their participation. Subjects reported no history of major medical or psychiatric conditions, and normal or corrected-to-normal vision. All procedures were approved by the Monash University Human Research Ethics Committee, and performed in accordance with the committee’s guidelines. Signed informed consent was obtained from all subjects prior to testing.

##### Stimuli

**FIGURE 2:**
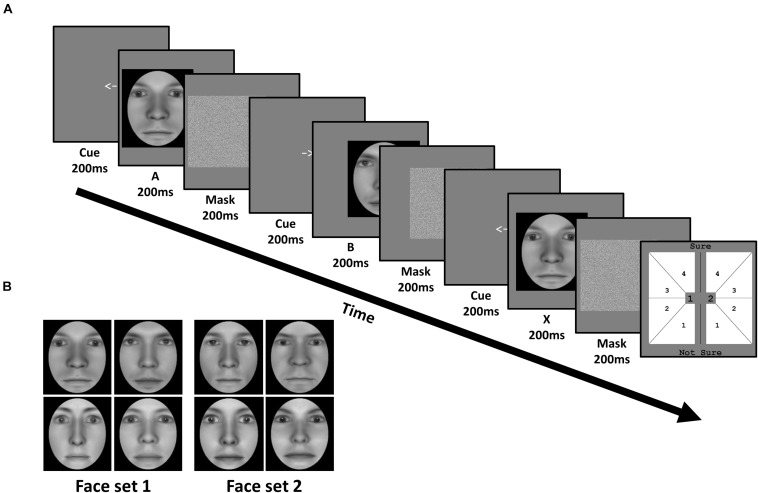
Task and stimuli of Experiment 1: Face identity matching. **(A)** In each trial, subjects viewed a sequence of three faces (A, B, and X). Each face was preceded by a cue corresponding to their on-screen displacement, and followed by a mask. Subjects judged whether the third face matched the identity of the first or second face, corresponding to 1 or 2 in the response screen (last panel), respectively. Subjects also simultaneously reported the confidence of their perceptual decision from 1 (not sure) to 4 (sure). **(B)** Subjects were trained on one face set, and untrained on the other, in a fully counterbalanced manner. Faces were generated using FaceGen software (v. 3.0; https://facegen.com/).

Four pairs of emotionally neutral, front-facing Caucasian faces (two female and two male) were generated using Facegen software (v. 3.0; Singular Inversions). All faces were converted to grayscale with a black oval mask applied to remove external features (e.g., ears), before normalizing each face pair on their luminance and contrast (Willenbockel et al., 2010). Within the oval mask, faces subtended 9.76 degrees of visual angle (dva) vertically, and 5.9 dva horizontally (Figure 2B). Morpheus software (Morpheus Development) was then used to morph both faces within a pair together by anchoring key features (e.g., eyes, nose), generating a morph continuum from 100% of one face (100%:0%), to 100% of the other face (0%:100%), in 2% increments.

##### Procedure

Subjects performed an unspeeded ABX task across 3 consecutive days. In each trial, we presented a given face pair, and required subjects to judge whether the third face (X) matched the first (A) or second (B) face’s identity (Figure 2A). Face A and face B were always derived from a morph between a given face pair. Subjects simultaneously provided both their perceptual response (X = A or X = B), and their confidence in this decision from ‘not sure (1)’ to ‘sure (4)’, via a single mouse click. X was always identical to either A or B, with equal probability. Subjects were encouraged to respond as accurately as possible, and to use the entire confidence scale. No feedback was provided.

All 3 faces (A, B, and X) were presented sequentially for 200 ms each. To avoid biasing fixations toward a particular facial region (e.g., eyes), each face had a random horizontal leftward or rightward displacement between 0.78 and 1.56 dva, relative to the screen’s center. The displacement of the first face (A) was randomly determined, with the displacement of the remaining 2 faces (B and X) being opposite to the preceding face, such that only 2 sequences were possible (left (A), right (B), left (X); right (A), left (B), right (X)). Before the presentation of each face, a central leftward- or rightward-pointing arrow (200 ms) reliably cued subjects to each faces’ subsequent displacement. After the presentation of each face, a Gaussian noise mask (200 ms) appeared which covered the spatial extent of the preceding face.

The task was programmed and run using the Psychophysics toolbox extension (Psychtoolbox-3) for Matlab (Brainard, 1997; Pelli, 1997). Stimuli were presented against a gray background on a 23-inch screen (1920x1080 pixels, 60 HZ refresh rate), which subjects viewed from a chinrest placed 75 cm away. Subjects were given the chance to take a short break after every 160 trials.

We estimated morph distance thresholds (%) corresponding to 75% accuracy (psychometric slope (β) = 0.1, lapse rate (δ) = 0.05, probability of a correct guessing response (γ) = 0.5), using quick estimation of threshold (QUEST; Watson and Pelli, 1983). QUEST implements an adaptive staircase procedure using Bayesian principles and provides the most probable estimation of stimulus threshold via a posterior distribution function (PDF). In each trial, morph threshold estimates (rounded to the nearest multiple of 2%, with a maximum of 100%) were halved and subtracted from, and added to, the morph midpoint (50%:50%) of a particular face pair to select A and B faces along the morph continuum. The morph distance of A (and B) had an equal probability of being the midpoint ± (threshold/2).

**FIGURE 3:**
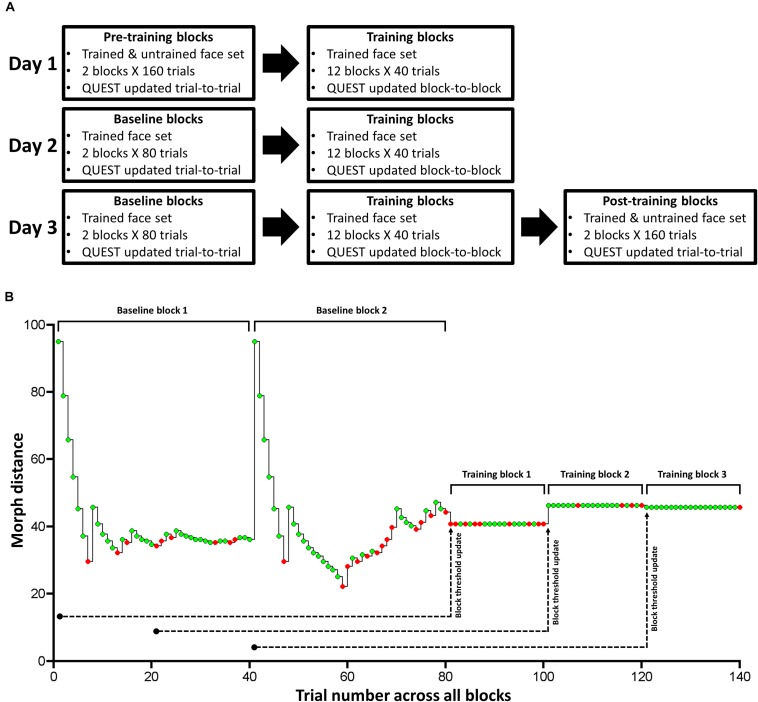
Task design for Experiment 1. **(A)** The paradigm consisted of pre-training, training, baseline, and post-training. We used QUEST to update stimulus difference (intensity) after each trial in pre-training, baseline, and post-training blocks. We trained subjects on a single face set (1 male and 1 female face pair) over 3 consecutive days in training and baseline blocks. In pre- and post-training blocks, which took place immediately before (Day 1) and after (Day 3) training, respectively, we tested subjects on the trained and untrained face set. **(B)** Exemplary time course of one of the two trained face pair’s morph distance and response accuracy for the 2 baseline blocks and the first 3 training blocks for one subject. Green and red circles correspond to correct and incorrect trials, respectively. Note that we do not show the time course for the other trained face pair. This time course clarifies our trial-to-trial and block-to-block QUEST protocol: We hold morph distance constant during each training block, and update it based on the previous 80 trials of a given training block. While we used 80 trials for our online QUEST updating procedure, we used the 20 trials within each training block (with a fixed morph distance) to compute: threshold, Type-I and II AUC, and the mean and variance of confidence ratings separately for correct and incorrect trials (offline data analysis). We used the same design for Experiment 2.

The experiment consisted of three primary phases: Pre-training, training, and post-training (Figure 3A). There was no explicit break between each phase within each experimental session. During pre- and post-training phases, subjects performed 2 separate blocks involving all 4 face pairs. Each block consisted of 4 randomly interleaved QUEST staircases, which followed the standard QUEST procedure (i.e., stimulus intensity updated from trial-to-trial). For each face pair, a single staircase (40 trials) was used to independently estimate the morph threshold that likely resulted in a discrimination accuracy of 75%. During these pre- and post-training blocks, we did not estimate Type-I or II AUC as these measures require multiple trials at a fixed stimulus intensity (see below).

Across 3 consecutive days, we trained half our subjects on face set 1, and the remaining half on face set 2. Each face set consisted of a pair of male and female faces (Figure 2B). During daily training, subjects performed 12 blocks. Each block consisted of 20 trials for one face pair and 20 trials for the other pair (i.e., a total of 40 trials/block), presented in a randomized order (Figure 3A).

In these training blocks, we held the morph distance between face A and B constant, so that we could reliably calculate Type-I and II AUC at a given morph distance for a particular face pair (Macmillan and Creelman, 2005). After each block, we adjusted the stimulus intensity for each face pair so that the percentage correct was kept at around 75% across blocks. To achieve this, we updated the QUEST estimate of threshold at the end of each block by supplying the history of each face pair’s morph distance and response accuracy over the last 80 trials. We defined the most informative PDF quantile obtained by QUEST as the threshold estimate for each face pair, and used it for their corresponding trials in the next training block.

For Day 1, we used the last 80 trials during the pre-training blocks for a given face pair to estimate its threshold for training block 1. Then, we used the last 60 pre-training trials and the 20 trials of training block 1 to update the threshold estimate for training block 2, and so on. For Day 2 and 3, we ran 2 baseline blocks of 80 trials (40 trials per trained face pair), in which we updated threshold from trial-to-trial using QUEST, before subjects completed the 12 training blocks (Figure 3A). Note that this use of a block-to-block QUEST updating procedure is quite unlike the standard use of QUEST, in which the threshold is updated from trial-to-trial, as we did for our baseline, pre- and post-training blocks. To make this point clear, we show an exemplar time course of morph distance and response accuracy for one face pair over the 2 baseline blocks and the first 3 training blocks, for one subject (Figure 3B).

##### Data analysis

###### Pre- and post-training

Pre- and post-training consisted of 2 blocks. Each block involved 4 randomly interleaved QUEST staircases, with a single staircase (40 trials) for each face pair (i.e., 2 pre-training and 2 post-training staircases per face pair). For each QUEST staircase, we updated the threshold estimate from trial-to-trial. To measure training effects, we assessed the morph thresholds for all 4 face pairs before and after 3 days of training, with subjects being trained on only 2 of these face pairs. For the 40 trials of each staircase, we defined the mode of its PDF as the threshold (Watson and Pelli, 1983). We then averaged staircase thresholds from the two corresponding face pairs to obtain thresholds for the trained and untrained face set for each subject.

###### Training

Daily training consisted of 12 blocks. Each block involved 20 trials for each trained face pair, which were presented in a randomized order. Within a given block, stimulus intensity for each face pair was held constant (Figure 3B). For each of the 12 training blocks, we separately calculated the following measures for each face pair: threshold, objective accuracy (Type-I AUC), metacognitive accuracy (Type-II AUC), mean confidence ratings for correct and incorrect trials, and the variance of confidence ratings for correct and incorrect trials.

To estimate the threshold for a face pair in a given training block of 20 trials, we used QUEST to construct a PDF from these trials, and defined its mode as the threshold (Watson and Pelli, 1983). Note that we only used 20 trials in our offline data analysis (i.e., threshold, Type-I and II AUC, and confidence ratings), but we used the previous 80 trials to update stimulus intensity in our online experiment (Figure 3B).

We calculated objective and metacognitive accuracy using a receiver operating characteristics (ROC) curve based on SDT. For objective accuracy, we constructed a Type-I ROC curve, which reflects the perceptual discriminability between face A and B independent of response criteria. Given we held stimulus intensity constant within training blocks, Type-I ROC curves were independently calculated on a block-by-block basis. To achieve this, we considered X = A trials as signal present trials, and X = B trials as signal absent trials. Hits and false alarms were then estimated by systematically varying the response criterion in 7 steps. Firstly, we regarded a response as a ‘hit’ when the signal was present, and subjects reported X = A with the highest confidence (4). Similarly, we regarded a response as a ‘false alarm’ when the signal was absent, and subjects reported X = A with the highest confidence (4). We then shifted the criterion to include X = A responses endorsed with a confidence of 3 and 4, and likewise classified responses as hits or false alarms depending on whether the signal was present or absent, respectively. We shifted the response criterion in this manner until we obtained hits and false alarms from the highest (4) to lowest (1) confidence ratings for X = A responses, and the lowest (1) to second highest (3) confidence ratings for X = B responses to obtain a ROC curve with 7 inflection points. We use the Area Under the ROC Curve, or Type-I AUC, as a non-parametric estimate of objective task accuracy (Macmillan and Creelman, 2005; Wilimzig et al., 2008; Kaunitz et al., 2016).

For metacognitive accuracy, we constructed a Type-II ROC curve, which quantifies the discriminability of correct and incorrect decisions independent of response criterion. We decided to implement Type-II ROC curves over meta-d’ (Maniscalco and Lau, 2012) as we found that the parametric assumptions of meta-d’ were not met when we examined the Type-I distributions of our participants (see Supplementary Material). As with Type-I ROC curves, Type-II ROC curves were also independently calculated on a block-by-block basis, given stimulus intensity was held constant within training blocks. To achieve this, we considered trials where perceptual decisions were correct (i.e., subjects reported X = A in X = A trials, and X = B in X = B trials) as signal present trials, and trials where perceptual decisions were incorrect as signal absent trials. Hits and false alarms were then estimated by systematically varying the response criterion in 3 steps. Firstly, we regarded a response as a ‘hit’ when a signal present trial was endorsed with the highest confidence (4), and a ‘false alarm’ when a signal absent trial was endorsed with the highest confidence (4). We then shifted the criterion to include responses endorsed with a confidence of 3 and 4, and likewise classified responses as hits or false alarms depending on whether the signal was present or absent, respectively. We shifted the criterion in this manner until we obtained hits and false alarms from the highest (4) to second lowest (2) confidence ratings to obtain a ROC curve with 3 inflection points. We use the Area Under the ROC Curve, or Type-II AUC, as a non-parametric estimate of metacognitive accuracy (Galvin et al., 2003; Wilimzig et al., 2008; Kaunitz et al., 2016).

For confidence, we separately calculated the mean and the variance of confidence ratings for correct and incorrect trials.

##### Statistical analysis

###### Pre- and post-training

To test whether post-training thresholds were significantly lower than pre-training thresholds for the trained and the untrained face set, we used one-tailed Wilcoxon signed-rank tests.

To estimate the degree of VPL transfer from the trained to untrained set for each subject, we defined a transfer index (TI) as [threshold improvement for the untrained set / threshold improvement for the trained set], with TI = 1 corresponding to complete VPL transfer, and TI = 0 to no transfer (Bi et al., 2010). We defined threshold improvement as [(Threshold_pre–training_ – Threshold_post–training_)/Threshold_pre–training_] × 100%. To test whether TI was significantly greater than or less than T1 = 0 and TI = 1, respectively, we used one-tailed one-sample *t*-tests.

###### Training

To investigate the effects of daily training on threshold, Type-I and II AUC, and the mean and variance of confidence ratings (separately for correct and incorrect trials), we performed separate linear mixed-effects analyses using lme4 package (Bates et al., 2015) within R software (R Foundation for Statistical Computing). We constructed a 2x3 nested mixed design, with trained face set (i.e., face set 1 or face set 2) as a between-subject variable, and daily training session (i.e., Day 1 to Day 3) as a within-subject variable. We modeled daily training session as a fixed effect. As random effects, we modeled an intercept for each face set to account for variances in learning effects between the sets, as well as a by-subject intercept and slope for daily training session to account for subject variability in learning effects, and the rate of these effects across training sessions. We performed likelihood ratio tests between the full model, as described above, and a reduced model, without daily training sessions modeled as a fixed effect, to obtain chi-squared statistics and associated *p*-values.

### Results

**FIGURE 4:**
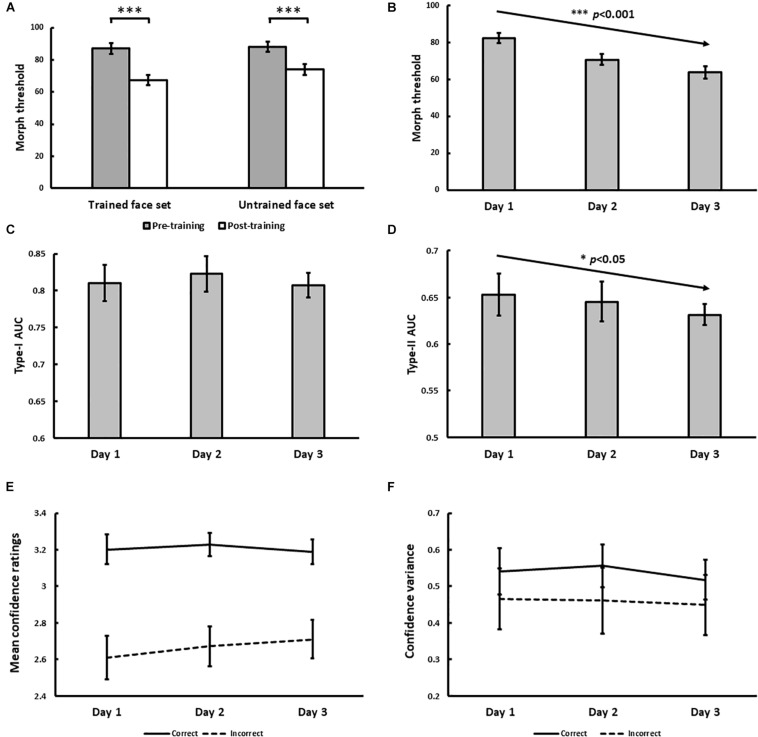
Results for Experiment 1. **(A)** Pre- and post-training effects measured as median morph threshold for trained and untrained face sets before (on Day 1) and after (on Day 3) training sessions. (^∗∗∗^ indicates *p* < 0.001). Threshold significantly decreased with training over 3 days **(B)**, which was accompanied by decreases in Type-II AUC (metacognitive accuracy, *p* = 0.015; **(D)** None of the following changed with training: Type-I AUC for objective accuracy **(C)**, confidence ratings for correct (solid) and incorrect (dashed) trials **(E)**, and the variance of confidence ratings for correct (solid) and incorrect (dashed) trials **(F)**. All 5 variables were independently estimated within each training block (see Exp. 1 Method). Error bars denote ± 1 within-subjects SEM (Cousineau, 2005).

The psychophysical results of Experiment 1 are displayed in Figure 4. From pre- to post-training, morph threshold significantly decreased for both the trained (*Z* = −3.51, *p* < 0.001) and the untrained (*Z* = −3.32, *p* < 0.001) face set (Figure 4A). Transfer index (see Method) was 0.67 (*SD* = 1.20), which was significantly greater than 0 (*p* = 0.011), but not less than 1 (*p* = 0.11). This suggests that with training, subjects successfully demonstrated identity-invariant VPL of face identity.

With daily training, morph threshold steadily decreased, confirmed by a significant main effect of training on threshold [χ^2^(1) = 27.06, *p* < 0.001; Figure 4B]. As intended by our QUEST procedure however, no main effect of training on objective accuracy (Type-I AUC) was observed [χ^2^(1) = 0.10, *p* = 0.75; Figure 4C]. Interestingly, metacognitive accuracy (Type-II AUC) decreased with training [χ^2^(1) = 4.52, *p* = 0.034; Figure 4D], arguing against improved metacognition as predicted by our single-stage model (Figures 1E,F). This decrease in metacognitive accuracy could not be simply attributed to biases in the use of confidence ratings, as we found no main effect of training on mean confidence ratings for correct [χ^2^(1) = 0.03, *p* = 0.87] and incorrect [χ^2^(1) = 1.29, *p* = 0.26] trials (Figure 4E), and no main effect of training on the variance of confidence ratings for correct [χ^2^(1) = 0.054, *p* = 0.46] and incorrect [χ^2^(1) = 0.24, *p* = 0.63] trials (Figure 4F).

In addition to the block-by-block analysis of Type-I and Type-II AUC reported above, we also tested the robustness of this finding by performing a secondary analysis where participants’ daily training trials (*n* = 480 trials) were pooled across training blocks. We used these pooled trials to compute a single daily estimate of Type-I and Type-II AUC for each participant using the same method described in our Data Analysis section, before testing for any training effects using repeated-measures ANOVAs. This secondary analysis revealed comparable findings to our primary analysis, namely a statistically significant decrease in Type-II AUC with face identity VPL, while Type-I AUC remained constant (see Supplementary Material).

### Experiment 2: Face Contrast VPL

#### Method

##### Subjects

Twenty subjects (14 female and 6 male, *M*_*age*_ = 24.9, *SD* = 5.24), who did not participate in Experiment 1, were recruited. All aspects of subject recruitment were the same as those for Experiment 1. Below, we describe the methodological differences between Experiment 1 and 2.

##### Stimuli

**FIGURE 5:**
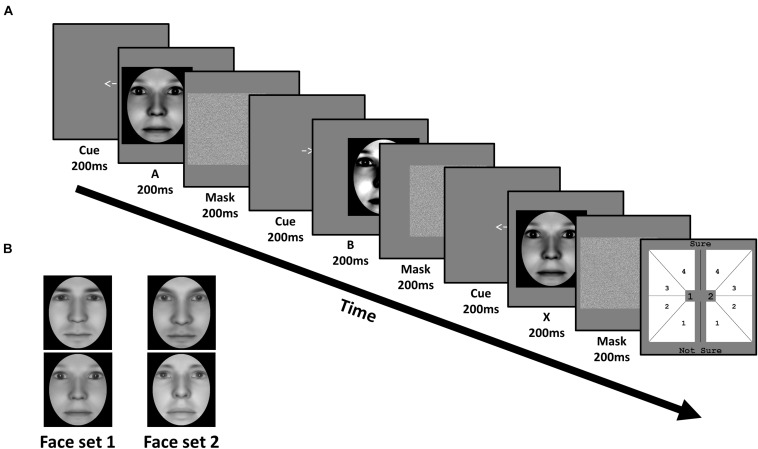
Task and stimuli of Experiment 2: Face contrast matching. **(A)** The task performed by subjects was identical to the task used in Experiment 1, except for the nature of the face stimuli. **(B)** Subjects were trained on one face set, and untrained on the other, in a fully counterbalanced manner. Faces were generated using FaceGen software (v. 3.0;https://facegen.com/).

Four emotionally neutral, front-facing Caucasian faces (2 female and 2 male) that differed in identity to the faces used in Experiment 1, were generated using FaceGen software. Using the same method as Experiment 1, each face was converted to grayscale, and a black oval mask applied (Figure 5B).

##### Procedure

The following summarizes the changes in Experiment 2. In each trial, we presented a face of the same identity as A, B, and X, and required subjects to judge whether the contrast of the third face matched that of the first or second face (Figure 5A). X was always identical to either A or B, with equal probability. We define the contrast of face A, B, and X as their normalized root mean square (nRMS) contrast. To obtain nRMS contrast, we calculated the standard deviation of luminance within the oval mask of each face, and normalized it by their mean luminance (set to 125 cd/m^2^ for all faces). We chose nRMS contrast based on its reliability in predicting human contrast sensitivity to natural images (Bex and Makous, 2002). We estimated nRMS contrast thresholds (in log scale) corresponding to 75% accuracy using QUEST (β = 3.5, δ = 0.05, and γ = 0.5). In each trial, we first converted contrast threshold estimates to linear scale (i.e., 10^*threshold*^), before halving the threshold and subtracting it from, and adding it to, the nRMS contrast midpoint (0.5 linear scale) for a particular face to derive the contrast values for A and B faces. The contrast of A (and B) had an equal probability of being the midpoint ± (threshold/2).

For both pre- and post-training phases, subjects were tested on all 4 faces. During training, we trained half our subjects on face set 1, and the other on face set 2. Each face set consisted of a male and female face (Figure 5B). In all 3 phases of our task design, each face was used in place of each face pair in Experiment 1.

##### Data and statistical analysis

Experiment 2 followed the same data and statistical analysis as Experiment 1, with the exception of the following changes. In both analyses, each face was used in place of each face pair in Experiment 1. Furthermore, estimates of contrast threshold for each face used the following QUEST parameters: β = 3.5, δ = 0.05 and γ = 0.5.

##### Data availability

The psychophysical dataset analyzed in Exp. 1 and 2, and the signal detection models generated in this study, are available in the Perceptual-learning-metacognition-study repository: https://github.com/DBenChen/Perceptual-learning-metacognition-study.

### Results

**FIGURE 6:**
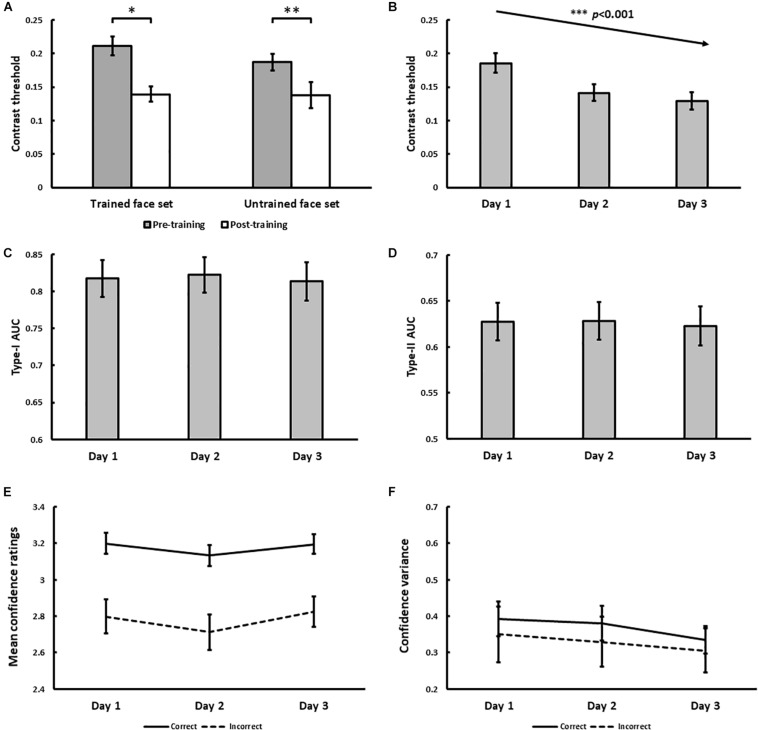
Results for Experiment 2. **(A)** Pre- and post-training effects measured as median contrast threshold for trained and untrained face sets before (on Day 1) and after (on Day 3) training sessions. (^*^ and ^∗∗^ indicate *p* < 0.05 and *p* < 0.01, respectively). Although threshold significantly decreased with training over 3 days **(B)**, none of the following changed with training: Type-I AUC for objective accuracy **(C)**, Type-II AUC for metacognitive accuracy **(D)**, confidence ratings for correct (solid) and incorrect (dashed) trials **(E)**, and the variance of confidence ratings for correct (solid) and incorrect (dashed) trials **(F)**. Error bars denote ± 1 within-subjects SEM (Cousineau, 2005).

The results of Experiment 2 are shown in Figure 6. From pre- to post-training, contrast threshold significantly decreased for both the trained (*Z* = −2.17, *p* = 0.015) and untrained (*Z* = −2.54, *p* = 0.006) face set (Figure 6A). Transfer Index was 0.62 (*SD* = 1.25), which was significantly greater than 0 (*p* = 0.02), but not less than 1 (*p* = 0.09). This suggests that with training, subjects successfully demonstrated identity-invariant VPL of face contrast.

With daily training, contrast threshold steadily decreased, confirmed by a significant main effect of training on threshold [χ^2^(1) = 12.62, *p* < 0.001; Figure 6B]. As intended by our QUEST procedure however, no main effect of training on objective accuracy (Type-I AUC) was observed [χ^2^(1) = 0.28, *p* = 0.60; Figure 6C]. Importantly, no main effect of training on metacognitive accuracy (Type-II AUC) was also found [χ^2^(1) = 0.34, *p* = 0.56; Figure 6D], consistent with improved metacognition as predicted by our single-stage model (Figures 1E,F). Similarly, we also found no main effect of training on mean confidence ratings for correct [χ^2^(1) = 0.003, *p* = 0.96] and incorrect [χ^2^(1) = 0.12, *p* = 0.73] trials (Figure 6E), and no main effect of training on the variance of confidence ratings for correct [χ^2^(1) = 2.16, *p* = 0.14] and incorrect [χ^2^(1) = 1.84, *p* = 0.18] trials (Figure 6F).

As with Experiment 1, we also tested the robustness of our AUC findings by performing a secondary analysis where participants’ daily training trials were pooled to compute a single daily Type-I and Type-II AUC estimate for each participant (in contrast to the block-by-block method of our primary analysis). This analysis revealed comparable results to the primary analysis of Experiment 2, namely both Type-I and Type-II AUC remaining constant with face contrast VPL (see Supplementary Material).

## Model II: Dual-Stage Noise Reduction Model

While the metacognitive accuracy result in Experiment 2 was consistent with our single-stage model, the metacognitive accuracy result in Experiment 1 was not. The primary reason is that the single-stage model derives Type-I and II AUC from the same signal, resulting in a highly correlated Type-I and II AUC (*r* = 0.99). To reproduce the dissociation between Type-I and II AUC in Experiment 1, it is necessary to decrease this correlation by considering a model where Type-I and II AUC are not derived from the same signal.

Given recent evidence from studies of metacognition (Pleskac and Busemeyer, 2010; Ratcliff and Starns, 2013; Cortese et al., 2016), it is reasonable to assume a first stage for perceptual decision-making, and a second stage for confidence judgements that inherits the signals used in perceptual decision-making, but also receives additional noise. Accordingly, we propose a different model that follows the same basic architecture and assumptions of our single-stage model (Figures 1A,B), with the following exception. Prior to assigning confidence ratings to internal response values, we added Gaussian noise (mean = 0, standard deviation = σ_*II*_, where σ_*II*_ > 0) to the internal responses. Confidence ratings were then assigned to the noised internal responses, using the same method as our single-stage model (Figure 1B). We term this model the ‘dual-stage model’.

**FIGURE 7:**
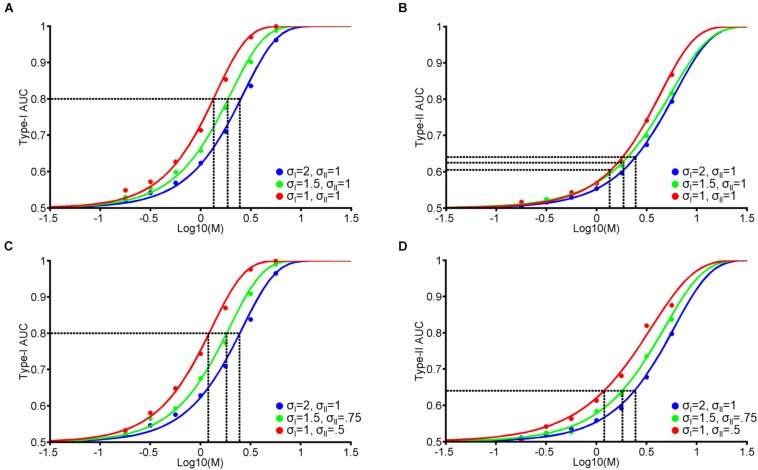
Dual-stage signal detection model: Psychometric functions. We added Gaussian noise (mean = 0, standard deviation = σ_*II*_) to the internal responses in our single-stage model (Figure 1) prior to assigning confidence ratings. **(A)** Hypothetical psychometric functions corresponding to Type-I AUC at different levels of σ_*I*_ noise (blue: σ_*I*_ = 2, green: σ_*I*_ = 1.5, and red: σ_*I*_ = 1) at constant noise for confidence judgements (σ_*II*_ = 1). Vertical dashed lines indicate thresholds at each level of noise to attain a Type-I AUC of 0.8 (horizontal dashed line). **(B)** Hypothetical Type-II AUC psychometric function with the same format as **(A)**. **(A,B)** Correspond to the results of Experiment 1: Face indentity VPL, where Type-I AUC remained constant **(A)** but Type-II AUC decreased **(B)** over 3 training days. **(C,D)** Type-I and II AUC psychometric functions where we modulated the noise associated with perceptual decision-making (blue: σ_*I*_ = 2, green: σ_*I*_ = 1.5, and red: σ_*I*_ = 1) and confidence judgements (blue: σ_*II*_ = 1, green: σ_*II*_ = 0.75, and red: σ_*II*_ = 0.5). **(C,D)** Correspond to the results of Experiment 2: Face contrast VPL, where both Type-I **(C)** and II **(D)** AUC remained constant over 3 training days.

In the dual-stage model, we consider the possibility that VPL reduces the amount of noise (σ_*II*_) added to internal responses prior to assigning confidence judgements, independent of the amount of noise reduction for perceptual decision-making (σ_*I*_). The key findings of Experiment 1 to be explained by this model are: (i) decreased sensory thresholds, (ii) constant Type-I AUC, and (iii) decreased Type-II AUC (Figures 4B,D). Figures 7A,B are exemplary modeling results that reproduce the psychophysical results of Experiment 1. For this, we needed to assume that VPL decreases the noise associated with perceptual decision-making (σ_*I*_), but not the noise associated with confidence judgements (σ_*II*_). Figures 7C,D show an example that reproduces the results of Experiment 2 (Figures 6B–D). To achieve this, we needed to assume that VPL decreases the noise for both perceptual decision-making and confidence judgements.

**FIGURE 8:**
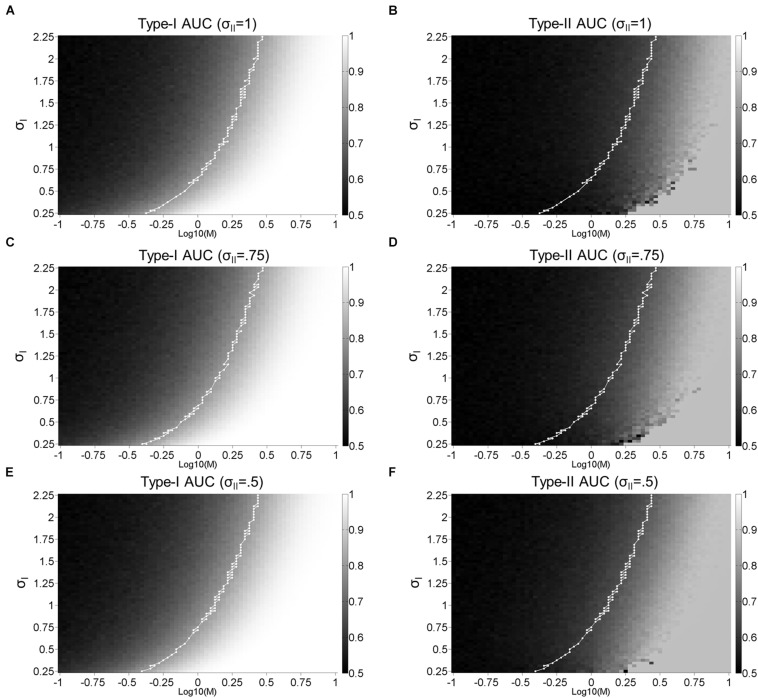
Dual-stage signal detection model: Surface plots. Surface plots for Type-I **(A,C,E)** and Type-II **(B,D,F)** AUC as a function of M and σ_*I*_ with σ_*II*_ = 1 **(A,B)**, σ_*II*_ = 0.75 **(C,D)**, and σ_*II*_ = 0.5 **(E,F)**. The white lines represent the points where Type-I AUC is around 0.8 (0.79–0.81). Type-I AUC is unaffected by the addition of noise (σ_*II*_) to confidence judgements as the first-order perceptual discriminations remain intact.

Figure 8 displays a more extensive parameter search in the dual-stage model and reveals how this model can disrupt the strong correlation between Type-I and II AUC. As the amount of σ_*II*_ increases from 0.5, 0.75, to 1, the correlation coefficient between Type-I and II AUC decreases from 0.96, 0.92, to 0.89 for Figures 8E vs. 8F, 8C vs. 8D, and 8A vs. 8B, respectively. Note that Type-I AUC is unaffected by the addition of noise (σ_*II*_) to confidence judgements as the first-order perceptual discriminations remain intact (i.e., proportion of hits:(hits+misses), and false alarms:(false alarms+correct rejections); Supplementary Figure S1).

In our Supplementary Material, we consider a model where VPL enhances the internal signal magnitude (i.e., a signal enhancement model; Gold et al., 1999), rather than decreasing the noise associated with perceptual decision-making (σ_*I*_). However, to reproduce the results of Experiment 1, the signal enhancement model predicts that VPL should *increase* the noise associated with confidence judgements (σ_*II*_). We found this physiologically implausible and therefore do not consider this model in further detail.

## General Discussion

We sought to investigate whether or not the metacognitive accuracy of perceptual decision-making could be improved by VPL, and whether learned stimulus complexity modulates this relationship. Across three consecutive days, subjects were trained to discriminate faces based on either their high-level identity (Exp. 1) or low-level contrast (Exp. 2). We measured objective and metacognitive accuracy as Type-I and II AUC, respectively. To control for objective accuracy during training, we devised a novel QUEST procedure which updates stimulus intensity only at the end of each training block, allowing the measurement of Type-I and II AUC within each block.

Holding objective accuracy constant across training days, we found that metacognitive accuracy decreased with face identity VPL (Figure 4D), which could not be attributed to changes in the mean or variance of confidence ratings (Figures 4E,F). According to our simple simulations, our face identity VPL result is inconsistent with a model where perceptual decision-making and confidence judgements are assumed to occur in a single stage (Figure 1). In the single-stage model, metacognitive accuracy is strongly correlated with objective accuracy (*r* = 0.99). As a result, Type-II AUC has to remain constant when Type-I AUC is held constant (Figures 1E,F). In fact, this prediction is consistent with the results of Experiment 2, in which Type-II AUC remained constant with face contrast VPL (Figure 6D).

To account for the decrease of metacognitive accuracy with face identity VPL (Figure 4D), we needed to dissociate Type-I and II AUC. We found that the correlation between Type-I and II AUC can be reduced through the introduction of a separate stage for confidence judgements, which inherits *noisy* signals from the perceptual decision-making stage (Figures 7, 8). If we assume that VPL reduces the noise associated with perceptual decision-making (σ_*I*_) but not confidence judgements (σ_*II*_), we could reproduce the pattern of results we obtained for Type-I and II AUC with face identity VPL (Figures 7A,B). Over the larger parameter search space, we confirmed that greater noise added before confidence judgements disrupted the strong correlation between Type-I and II AUC. This result is expected from the architecture of the dual-stage model and what Type-I and II AUC are supposed to measure: Type-I AUC is supposed to measure the quality of perceptual discrimination independent of metacognition. Type-II AUC is supposed to measure the accuracy of metacognition, which should degrade if the confidence judgment stage receives noisy perceptual signals.

Taken together, our results can be interpreted as demonstrating that when subjects improve the discrimination of high-level face identity, they do not improve conscious accessibility to the learned information supporting their enhanced perceptual decision-making. Conversely, conscious accessibility to such information is improved after training subjects to discriminate low-level face contrast. To our knowledge, this study is the first to obtain such a dissociative finding. If one presupposes that the processing of high-level visual properties should be more strongly associated with the conscious accessibility of information, our findings may appear counterintuitive. However, there are some hints in the literature (Hochstein and Ahissar, 2002; Ahissar and Hochstein, 2004) that are consistent with our findings, where the VPL of high-level visual properties are proposed to be mediated by unconscious mechanisms, while the VPL of lower-level properties are proposed to involve both conscious and unconscious mechanisms. If this is the case, this may have manifested as improvements in conscious accessibility with low-level face contrast VPL, but not high-level face identity VPL.

In the context of current views of metacognition, our key face identity VPL finding is not consistent with single-stage models for perceptual decision-making and confidence judgements (Galvin et al., 2003; Kepecs et al., 2008; Sanders et al., 2016). As we have demonstrated (Figure 1), such models should predict a strong correlation between Type-I and II AUC, and is therefore inconsistent with our face identity VPL result. With face contrast VPL on the other hand, metacognitive accuracy improved, which is consistent with single-stage perceptual decision-making and confidence models (Galvin et al., 2003; Kepecs et al., 2008; Sanders et al., 2016). Although one possible interpretation of our findings as a whole is that low-level VPL improves metacognition while high-level VPL does not, this requires further research to address whether these findings can be generalized to other VPL paradigms. Critical to such investigations is the use of our VPL protocol, which fixes objective accuracy by updating QUEST from block-to-block, allowing training effects on metacognitive accuracy to be reliably measured.

Furthermore, our protocol also addresses a key limitation within the VPL literature. VPL is generally assumed to produce knowledge of the learned stimuli that is independent of conscious experience (Fahle and Poggio, 2002). However, this conclusion has been largely inferred using objective measures of VPL performance (Manns and Squire, 2001; Fahle and Daum, 2002), rather than from subjective measures of VPL performance (e.g., confidence ratings). By measuring subjective confidence judgements and holding objective accuracy constant, we found that face contrast VPL improved metacognitive accuracy, suggesting subjects were conscious rather than non-conscious of the knowledge guiding their perceptual decision-making. Thus, our protocol can be used in future studies to more reliably investigate the relationship between VPL and consciousness.

In considering our study’s limitations, we acknowledge that the simulated psychometric functions of our models, which estimated performance (Type-I and II AUC) at a single stimulus difference, may not fully concur with the empirical estimation of the full psychometric function (Shen, 2013). Future studies should therefore seek to measure the full psychometric function, which may provide further insights into the effects of training on metacognition (e.g., changes in the slope of Type-II psychometric functions) which could not be discerned in the present study. However, if such studies were to be performed, we would predict that our conclusions here would remain unchanged, with metacognition improving with the VPL of face contrast but not face identity. Furthermore, although confidence ratings have been widely used as an index of conscious information accessibility (e.g., Galvin et al., 2003; Fleming et al., 2010), the relationship between confidence ratings and conscious accessibility remains controversial (Charles et al., 2013; Rausch and Zehetleitner, 2016). It would therefore be of interest for future research to attempt to replicate our present findings using alternative measures of consciousness, such as post-decision wagering (Sandberg et al., 2010).

In conclusion, we found evidence suggesting that conscious access to the information supporting perceptual decision-making is improved by the VPL of a low- but not high-level face property. Beyond VPL, our study can open new avenues to explore the relationship between metacognition and other learning paradigms, such as artificial grammar learning (Scott et al., 2014), and perceptual learning in non-visual modalities (Ahissar et al., 2009). Understanding the relationship between learning and consciousness in turn, constrains how our conscious experience is shaped by learned information, a central question in cognitive neuroscience (Bayne et al., 2009).

## Ethics Statement

All procedures were approved by the Monash University Human Research Ethics Committee, and performed in accordance with the committee’s guidelines. Signed informed consent was obtained from all subjects prior to testing.

## Author Contributions

BC and NT developed the study concept and design, and drafted the manuscript. BC performed the testing, data collection, data analysis, interpreted the results under the supervision of NT, and prepared the Figures 1–5. MM provided comments on the manuscript. All authors approved the final version for submission.

## Conflict of Interest Statement

The authors declare that the research was conducted in the absence of any commercial or financial relationships that could be construed as a potential conflict of interest.
