# Supplementary material for: Metacognitive Accuracy Improves With the Perceptual Learning of a Low- but Not High-Level Face Property
Source: Front Psychol. 2019 Jul 24;10:1712. doi: 10.3389/fpsyg.2019.01712 (PMC6667671; doi:10.3389/fpsyg.2019.01712)
Supplement: Supplementary file 1 [file Data_Sheet_1.docx]

**Supplementary information**

*Pooled Type-I and Type-II AUC analysis*

In the main text analysis for Experiments 1 and 2, we derived Type-I and Type-II AUC on a block-to-block basis, given our experimental design which held stimulus intensity constant within each training block. Here, we report an alternative analysis where we instead pooled each participant’s trials (i.e., 480 trials) across all training blocks to derive their individual Type-I and Type-II AUC for each day.

Under this trial-pooling approach, a repeated-measures ANOVA revealed a significant decrease in Type-II AUC with face identity VPL (*F*(2, 38) = 5.24, *p* = 0.01), but not face contrast VPL (*F*(2, 38) = 0.15, *p* = 0.86). Importantly, a repeated-measures ANOVA did not reveal a significant change in Type-I AUC with either face identity VPL (*F*(2, 38) = 0.69, *p* = 0.51) or face contrast VPL (*F*(2, 38) = 0.55, *p* = 0.58). Together, these results replicate the findings we report in the main text. The results for these new analyses are displayed below for Experiment 1 and 2 (Fig. S1 & S2).


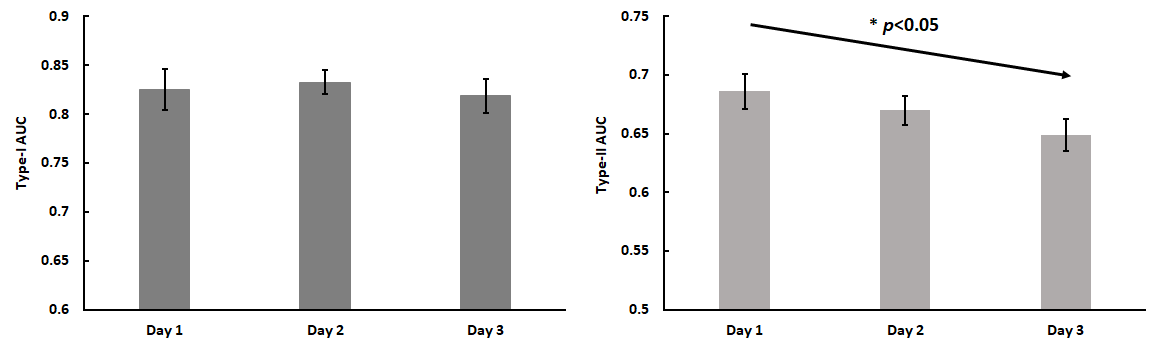
**Figure S1: Mean Type-I AUC (left) and Type-II AUC (right) with Face Identity VPL across Day 1-3.** Error bars represent ± 1 SEM.


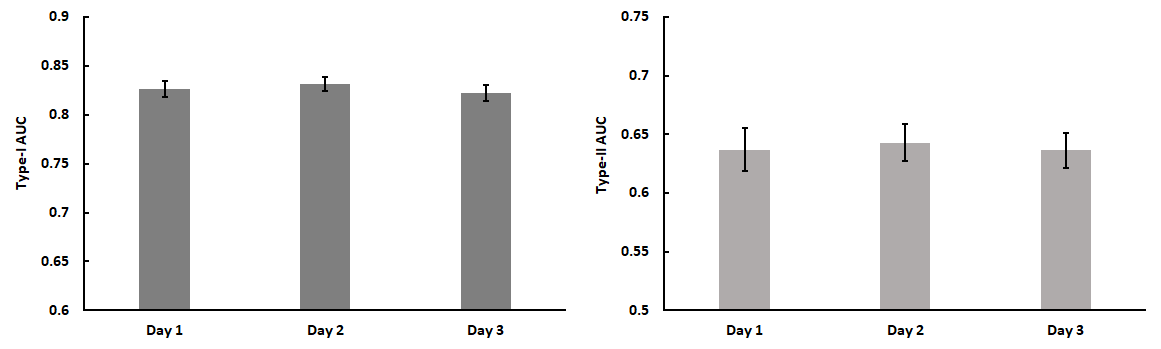
**Figure S2: Mean Type-I AUC (left) and Type-II AUC (right) with Face Contrast VPL across Day 1-3.** Error bars represent ± 1 SEM.

*Meta-d’ analysis*

Here, we report an alternative analysis using meta-d’ rather than Type-II AUC to investigate the relationship between metacognition and VPL in Experiment 1 and 2. First, we pooled each participant’s trials across all daily training blocks, and then individually calculated their daily meta-d’ for both trained faces using the toolbox provided by Maniscalco and Lau (2012). We then averaged the meta-d’ of both trained faces to obtain each participant’s daily meta-d’. A repeated-measures ANOVA revealed no significant change in meta-d’ with either Face Identity VPL (*F*(2, 38) = 1.17, *p* = .32) or Face Contrast VPL (*F*(2, 38) = 1.43, *p* = .25). These results are displayed in Fig. S3 below.


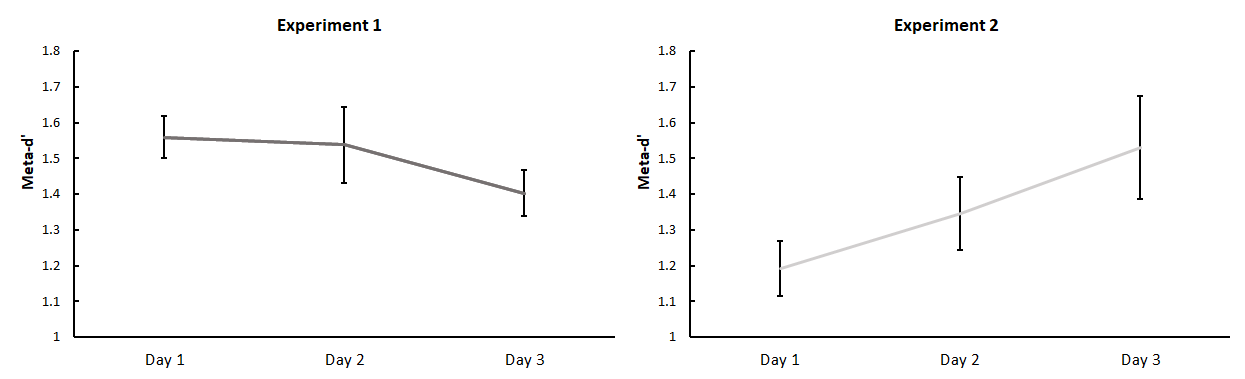
**Figure S3: Mean meta-d’ with Face Identity VPL (left) and Face Contrast VPL (right) across Day 1-3.** Error bars represent ± 1 SEM.

While this finding might appear inconsistent with our main findings, we found that the statistical assumption for meta-d’ was not met. Meta-d’ assumes that both ‘signal’ and ‘noise’ distributions to share equal variance (Maniscalco & Lau, 2012), which should result in symmetric ROC curves with respect to the diagonal (Macmillan & Creelman, 2005). As shown below, Type-I ROC curves in both Experiment 1 and 2 were largely asymmetrical (Fig. S4 & S5).


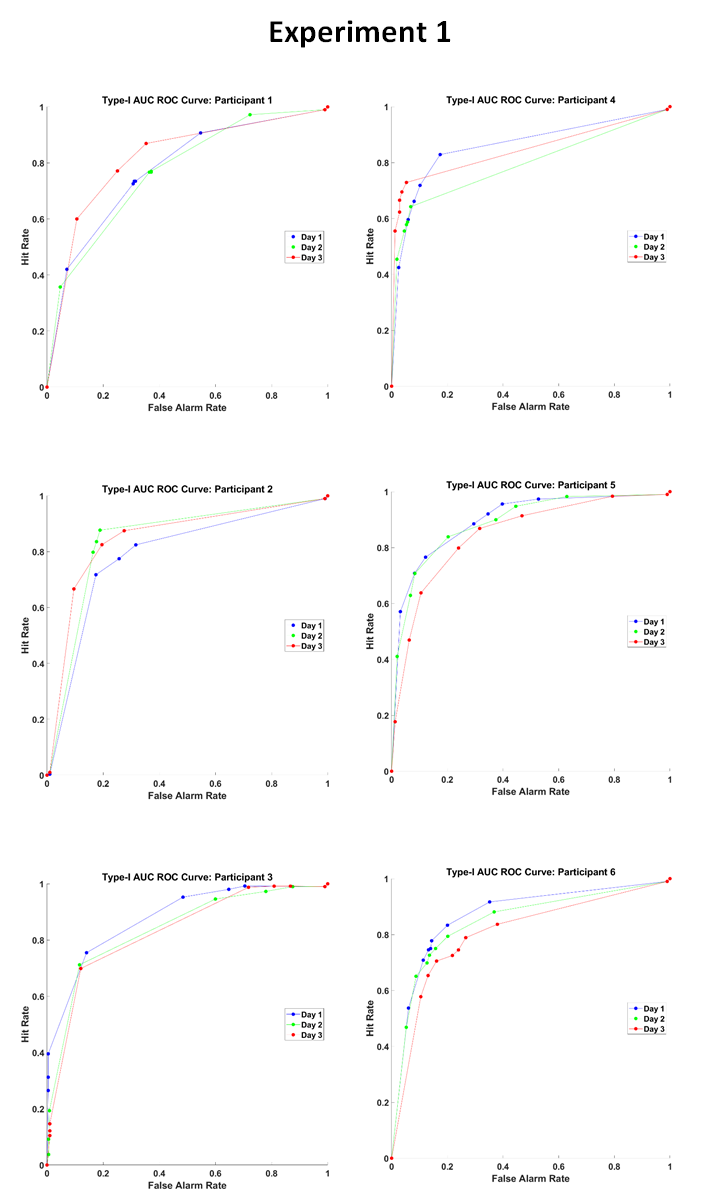
**Fig. S4: Type-I AUC ROC Curves for each participant in Experiment 1 (Face Identity VPL) from Day 1-3**. Each point from the lowest Hit and False Alarm Rate onwards in the Type-I AUC ROC Curves represents increasing confidence in an X=A perceptual decision.


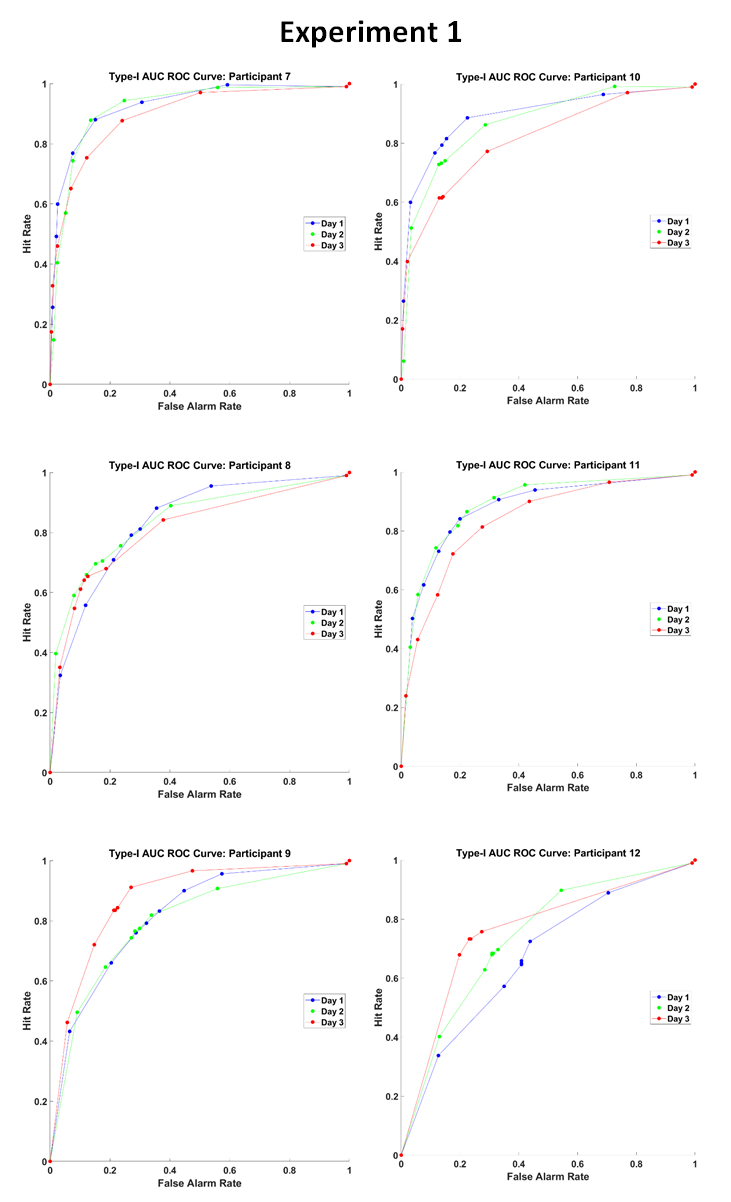


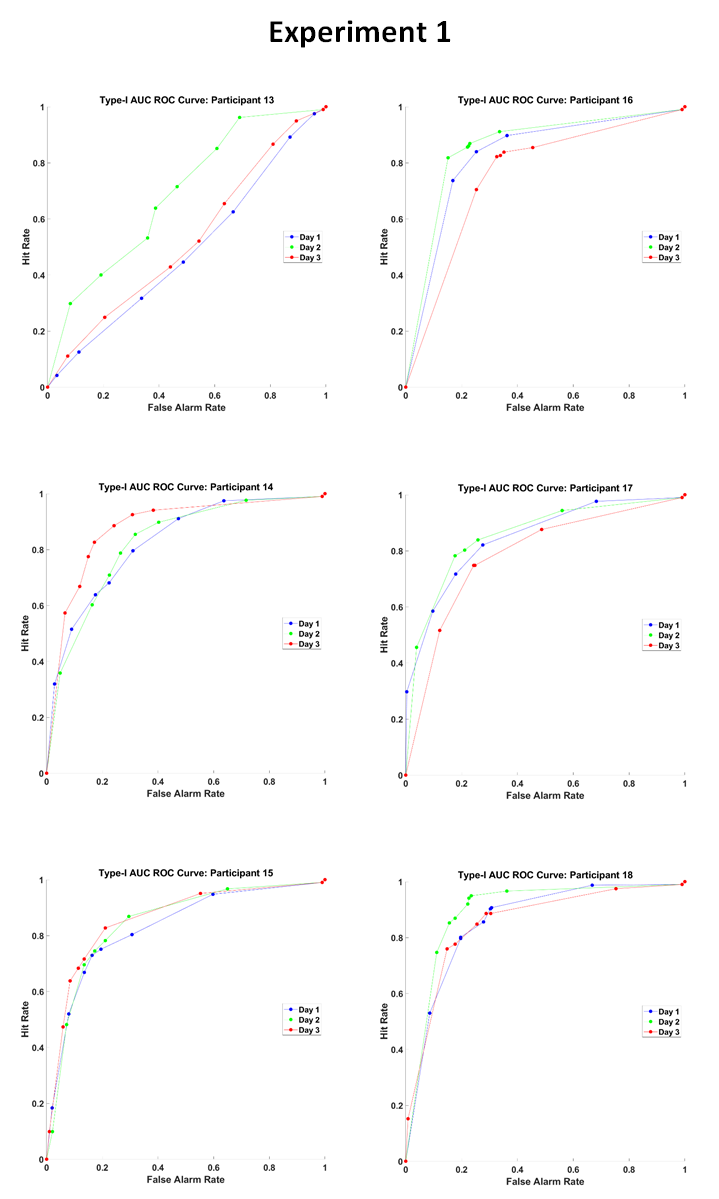


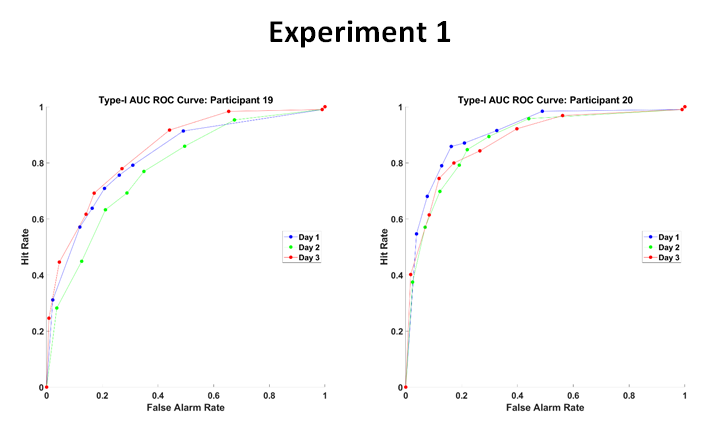


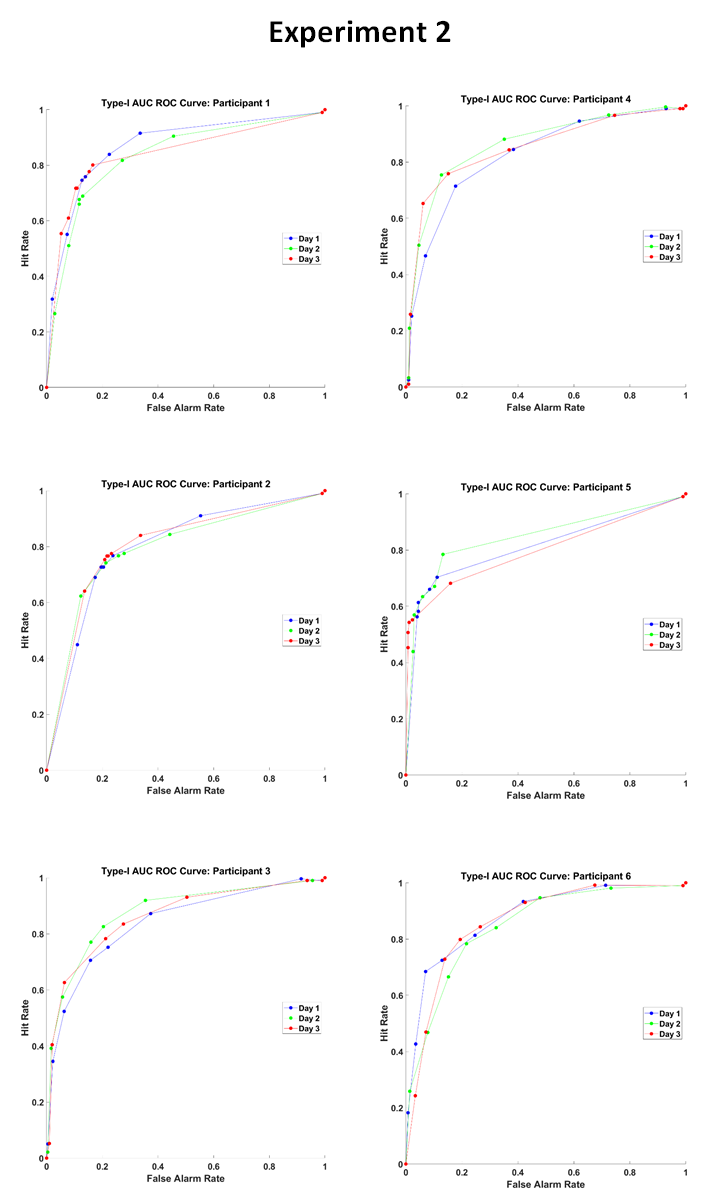
**Fig. S5: Type-I AUC ROC Curves for participants in Experiment 2 (Face Contrast VPL) from Day 1-3**. Each point from the lowest Hit and False Alarm Rate onwards in the Type-I AUC ROC Curves represents increasing confidence in an X=A perceptual decision.


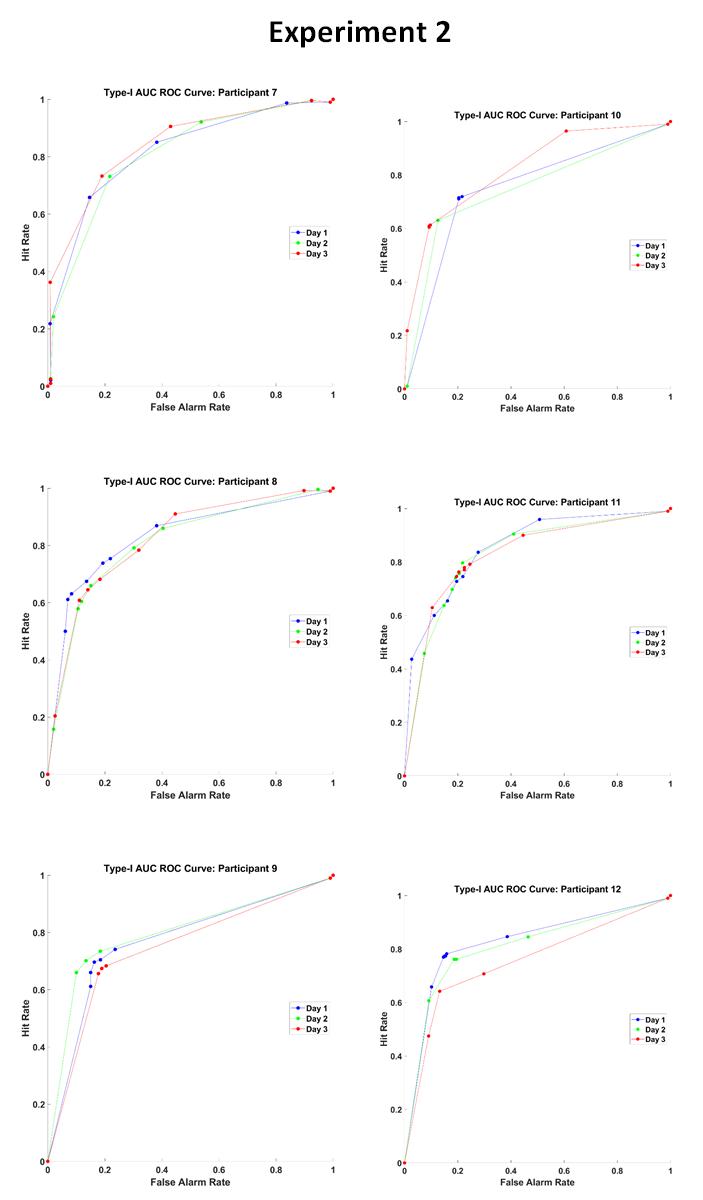


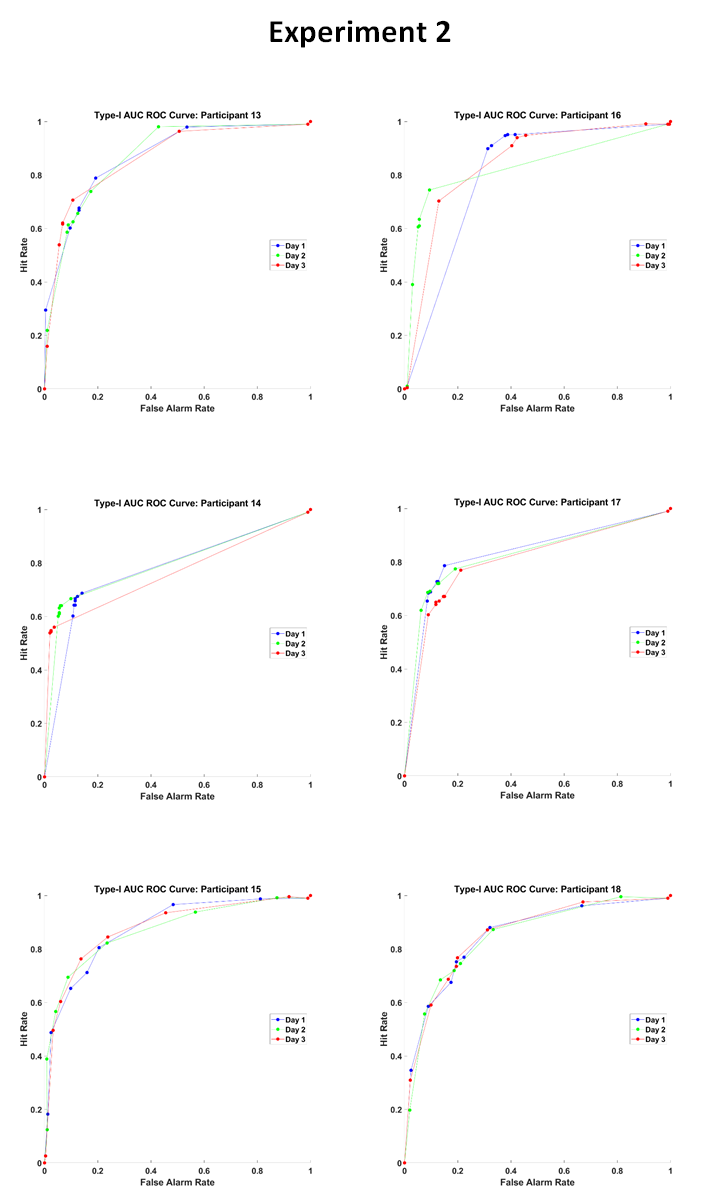


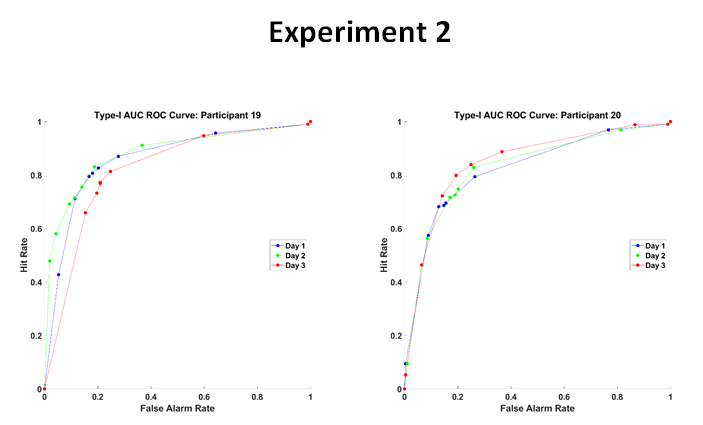


*Signal enhancement signal detection theory-based models*

In the main text and Figs. 1, 7 and 8, we did not provide information about our efforts to simulate signal enhancement models of visual perceptual learning (VPL; Gold et al., 1999). We did not pursue this model as the conclusion of this model did not offer a physiologically plausible account of our face identity and contrast VPL findings (Fig. 2 & 3). It is plausible however, that a model which modifies some of our assumptions described below may be able to account for our findings and provide physiologically plausible mechanisms.

Based on signal detection theory (SDT; Macmillan & Creelman, 2005), we constructed generative models that allow for the generation of internal responses that are dependent on stimulus intensity and the level of VPL. For internal responses, we assume two generative Gaussian distributions, with one corresponding to internal responses for face A trials, and the other corresponding to internal responses for face B trials (Fig. S6A & S7A). We define the distance between the means of the two distributions as M+α, with M relating to stimulus intensity, and the means of the face A and B distributions as (M+α)/2 and -(M+α)/2, respectively. As X=A trials and X=B trials occurred with equal probability in our 2AFC ABX task, we can make the following two reasonable assumptions in our models. Firstly, that both face A and B distributions have equal variance (σ_I_), and secondly, that the response criterion used for perceptual decision-making is unbiased (i.e., an optimal criterion value of zero).
From the generative models, we obtained around 5000 internal responses for face A and B distributions, respectively, by randomly allocating 10000 trial outcomes to either distribution with equal probability. If an internal response from either distribution occurred to the left of the decision criterion, we assigned a perceptual response of face B (i.e., X=B), otherwise a perceptual response of face A was assigned (i.e., X=A). From these, we classified these perceptual responses as hits, misses, false alarms, or correct rejections, in line with SDT.
 Before assigning confidence ratings (1-4) to perceptual responses, we added Gaussian noise (mean = 0, standard deviation = σ_II_), to both face A and B distributions. When σ_II_ = 0, it corresponds to what we term our ‘single-stage signal enhancement model’, where perceptual decision-making and confidence judgements occur simultaneously (Fig. S6; Galvin et al., 2003; Kiani et al., 2009, 2014). When σ_II_ > 0 however, it corresponds to what we term our ‘dual-stage signal enhancement model’, where confidence judgements occur after perceptual decision-making, and inherits the signals from this prior stage (Fig. S7 & S8; Cortese et al., 2016; Pleskac & Busemeyer, 2010; Ratcliff & Starns, 2013).
 After the addition of noise, we flipped both face A and B distributions at the perceptual response criterion, and calculated the 25^th^, 50^th^, and 75^th^ percentiles of the cumulated distributions, before accordingly assigning confidence ratings (1-4) in equal proportion based on these quantiles (Fig. S6B & S7B). From these perceptual responses and confidence ratings, we can compute Type-I and II area under the curve (AUC) (see Data analysis in main text).

In summary, our single- and dual-stage signal enhancement models are generative with up to 4 free parameters (M, α, σ_I_ & σ_II_). For a given σ_I_ and α (single-stage model), or σ_I_, σ_II_ and α (dual-stage model), we can obtain psychometric functions for Type-I and II AUC as a function of stimulus intensity (M). In our main experiment, our noise reduction models defined VPL as decreases in σ_I_. Here, our signal enhancement models instead defines VPL as increases in α (i.e., the distance between face A and B distributions; Gold et al., 1999), with σ_I_ remaining constant. In our main Experiments, our QUEST staircase method kept subjects’ Type-I AUC around 0.8 with training. Thus, our simulations were centred on the model parameters that would produce Type-I AUC around 0.8, and the corresponding Type-II AUC produced at these parameter values.
 For our single-stage signal enhancement model, we define training effects on perceptual decision-making and confidence judgements as increases in α. From these simulations, two properties of our single-stage model can be discerned. Firstly, the effects of training or VPL on objective accuracy can be readily explained by increases in α (Fig. S6D). Increasing α predominantly shifts the Type-I psychometric function to the left, resulting in a decrease in the stimulus intensity (M) needed to maintain a constant Type-I AUC around 0.8 (Fig. S6C). Secondly, whenever objective accuracy improves with VPL, metacognitive accuracy invariably improves (Fig. S6F). This corresponds to a change in the Type-II psychometric function, resulting in a constant Type-II AUC when stimulus intensity is decreased to maintain Type-I AUC with VPL (Fig. S6E).

For our dual-stage signal enhancement model, we define training effects on perceptual decision-making as increases in α, and training effects on confidence judgements as increases in σ_II_. From these simulations, we find that as with our single-stage model, the effects of VPL on objective accuracy can be explained by increases in α. As α increases, the Type-I psychometric function shifts leftward (Fig. S7C & S8A), resulting in a decrease in the stimulus intensity (M) needed to maintain a constant type-I AUC around 0.8 (Fig. S7D & S8B-D). Unlike our single-stage model however, our dual-stage model permits type-II AUC to either improve or not improve with VPL, depending on the effects of VPL on the noise associated with confidence judgements (σ_II_). If σ_II_ fails to decrease with VPL, then the Type-II psychometric function changes (Fig. S7E), resulting in constant Type-II AUC when stimulus intensity is decreased to maintain Type-I AUC (Fig. S7F). This corresponds to an improvement of both objective and metacognitive accuracy with VPL. However, if σ_II_ increases with VPL, then the Type-II psychometric function remains largely static (Fig. S8E), resulting in a reduction of Type-II AUC when stimulus intensity is decreased to maintain Type-I AUC (Fig. S8F-H). This corresponds to an improvement of objective accuracy with VPL, but not metacognitive accuracy.
 Although our dual-stage signal enhancement model can account for our face identity and contrast VPL findings, it offers physiologically implausible mechanisms. Within this model, non-improved metacognition is a result of VPL increasing the noise associated with confidence judgements (σ_II_), while improved metacognition is a result of VPL failing to change σ_II_. In contrast, our dual-stage noise reduction model (see main text) offers a more parsimonious and plausible account of improved (decreasing σ_II_) and non-improved (constant σ_II_) metacognition with VPL.

**Figure S6: Single-stage signal enhancement model architecture and model results simulating improved objective and metacognitive performance**. (**A**) Internal response distributions for face A (solid and dashed dark grey) and B (solid and dashed light grey) trials, with the difference in both distribution means (dark and light grey vertical lines) defined as M+α, with M corresponding to stimulus intensity. We assume the following: both distributions are Gaussian with equal variance (σ_I_), α increases with training, and an unbiased perceptual response criterion with a value of zero (black vertical line). From this criterion, we classify perceptual responses as hits (solid dark grey), misses (dashed dark grey), correct rejections (solid light grey), or false alarms (dashed light grey). Critically, no Gaussian noise was added to both distributions prior to assigning confidence judgements (σ_II_ = 0). (**B**) Flipped face A and B distributions at the perceptual response criterion. We assigned corresponding confidence ratings (1-4) according to set percentiles derived from the cumulated distributions (**C** & **E**). Training effects on hypothetical psychometric functions corresponding to Type-I (**C**) or II (**E**) area under the curve (AUC) as a function of stimulus intensity across Day 1, 2, and 3 (Blue, green, and red, respectively in **C** & **E**). Vertical dashed lines in **C** & **E** represent the decreasing stimulus thresholds needed to maintain a fixed Type-I AUC of 0.8 (horizontal dashed line; **C**), and the corresponding Type-II AUC at these thresholds (horizontal dashed line; **E**). Coloured points represent the outputs of our model, and the coloured lines represent the fitted psychometric function. (**D** & **F**) Surface plots illustrating Type-I (**D**) or II (**F**) AUC as a function of α and M parameters of our model, with constant σ_I_ equivalent to our hypothetical psychometric functions. Dashed blue, green, and red lines correspond to the Day 1, 2, and 3 α parameters, respectively, of our hypothetical psychometric functions (**C** & **E**). The white line in **D** & **F** represents the α and M parameters corresponding to Type-I AUC around 0.8 (.79-.81).


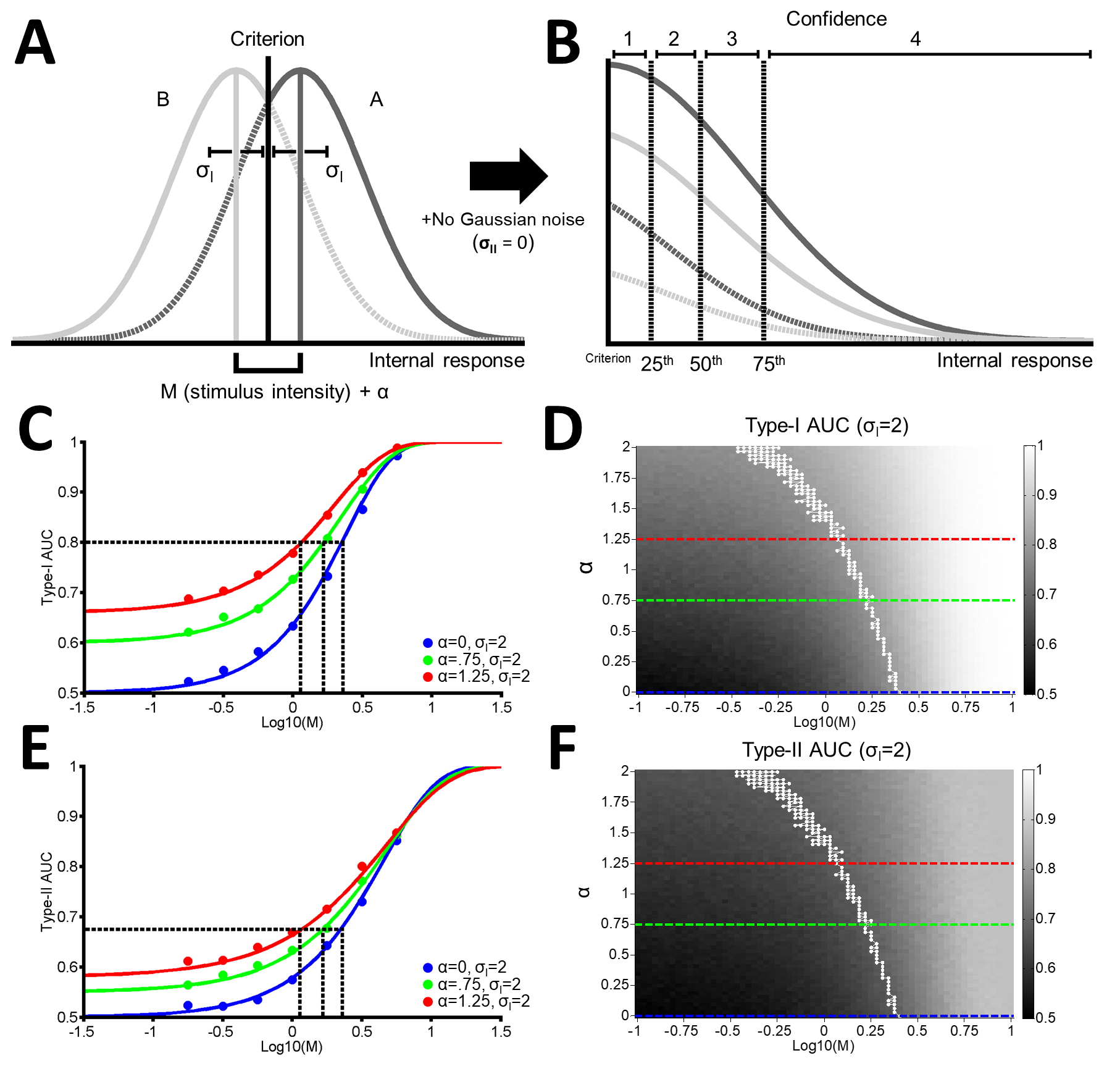


**Figure S7: Dual-stage signal enhancement model architecture and model results simulating improved objective and metacognitive performance**. (**A**) Internal response distributions for face A (solid and dashed dark grey) and B (solid and dashed light grey) trials, with the difference in both distribution means (dark and light grey vertical lines) defined as M+α, with M corresponding to stimulus intensity. We assume the following: both distributions are Gaussian with equal variance (σ_I_), α increases with training, and an unbiased perceptual response criterion with a value of zero (black vertical line). From this criterion, we classify perceptual responses as hits (solid dark grey), misses (dashed dark grey), correct rejections (solid light grey), or false alarms (dashed light grey). Critically, Gaussian noise was added to both distributions prior to assigning confidence judgements (σ_II_ > 0), which remained constant with training to simulate improved metacognition. (**B**) Flipped face A and B distributions at the perceptual response criterion. We assigned corresponding confidence ratings (1-4) according to set percentiles derived from the cumulated distributions. (**C** & **E**) Training effects on hypothetical psychometric functions corresponding to Type-I (**C**) or II (**E**) area under the curve (AUC) as a function of stimulus intensity across Day 1, 2, and 3 (Blue, green, and red, respectively in **C** & **E**). Vertical dashed lines in **C** & **E** represent the decreasing stimulus thresholds needed to maintain a fixed Type-I AUC of 0.8 (horizontal dashed line; **C**), and the corresponding Type-II AUC at these thresholds (horizontal dashed lines; **E**). Coloured points represent the outputs of our model, and the coloured lines represent the fitted psychometric function. (**D** & **F**) Surface plots illustrating Type I (**D**) or II (**F**) AUC as a function of α and M model parameters, with constant σ_I_ and σ_II_ equivalent to our hypothetical psychometric functions. Dashed blue, green, and red lines correspond to the Day 1, 2, and 3 α parameters, respectively, of our hypothetical psychometric functions (**C** & **E**). The white line in **D** & **F** represents the α and M parameters corresponding to Type I-AUC around 0.8 (.79-.81).


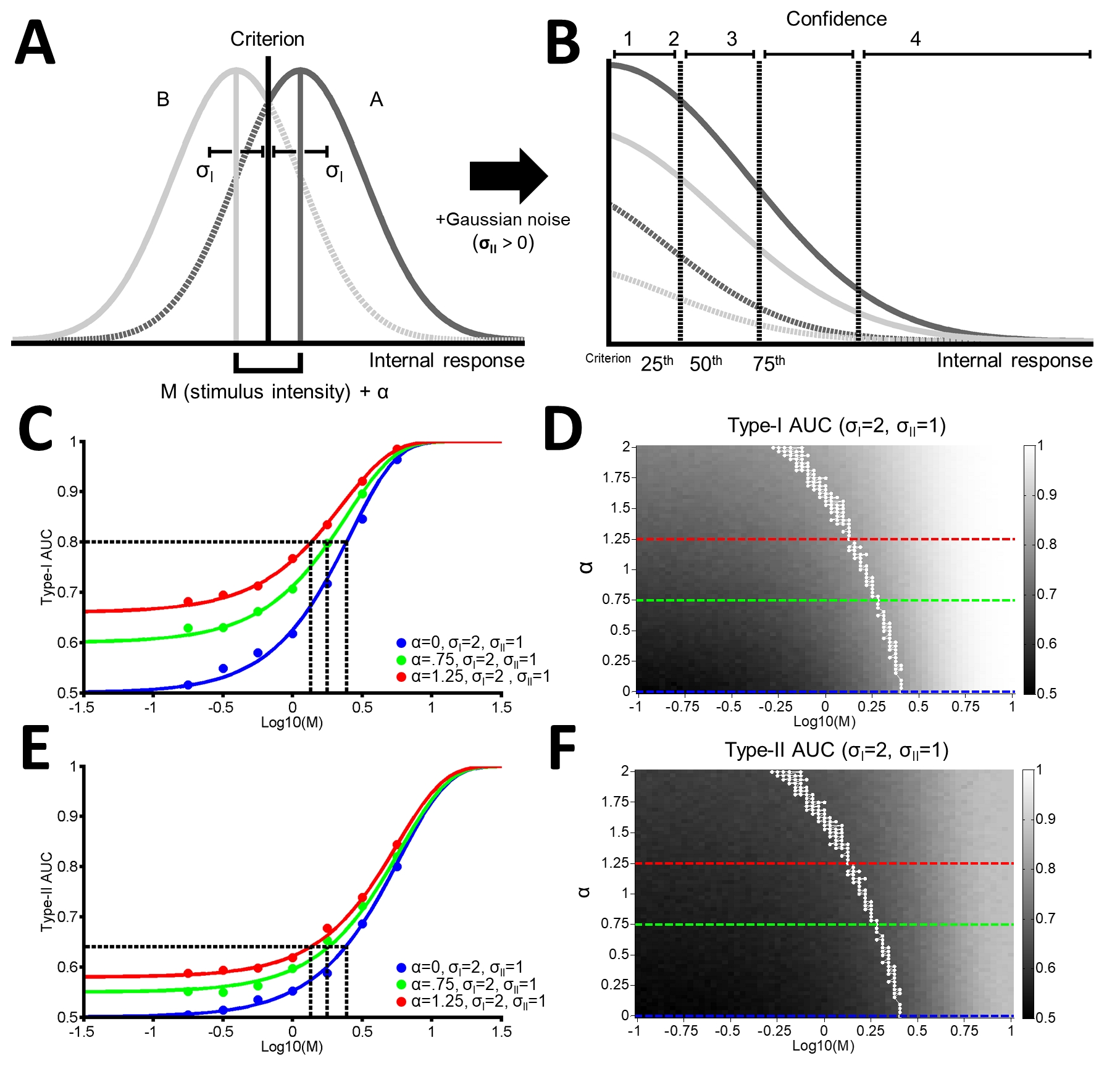


**Figure S8: Dual-stage signal enhancement model results simulating improved objective performance and non-improved metacognitive performance**. To simulate non-improved metacognition, the Gaussian noise added to both face A and B distributions (σ_II_) increased with training. (**A** & **E**) Training effects on hypothetical psychometric functions corresponding to Type-I (**A**) or II (**E**) area under the curve (AUC) as a function of stimulus intensity across Day 1, 2, and 3 (Blue, green, and red, respectively in **C** & **E**). Vertical dashed lines in **A** & **E** represent the decreasing stimulus thresholds needed to maintain a fixed Type-I AUC of 0.8 (horizontal dashed line; **C**), and the corresponding Type II AUC at these thresholds (horizontal dashed lines; **E**). Coloured points represent the outputs of our model, and the coloured lines represent the fitted psychometric function. (**B-D** & **F-H**) Surface plots illustrating Type-I (**B**-**D**) or II (**F-H**) AUC as a function of α and M model parameters, with constant σ_I_ and increasing σ_II_ equivalent to our hypothetical psychometric functions. Dashed blue (**B** & **F**), green (**C** & **G**), and red (**D** & **H**) lines correspond to the Day 1, 2, and 3 α parameters, respectively, of our hypothetical psychometric functions. The white line in **B**-**D** & **F-H** represents the α and M parameters corresponding to Type-I AUC around 0.8 (.79-.81).


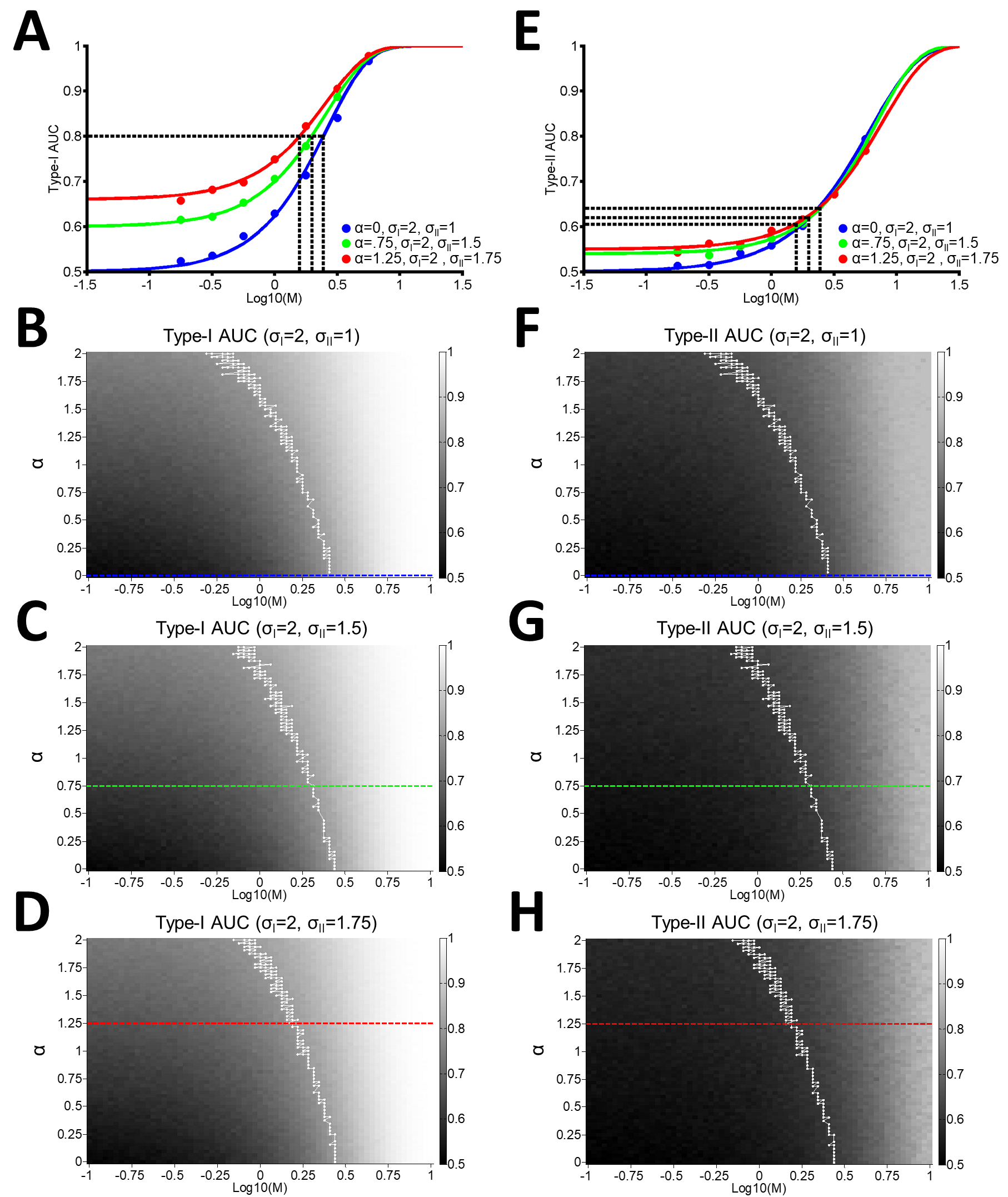


**References**

Cortese, A., Amano, K., Koizumi, A., Kawato, M., and Lau, H. (2016). Multivoxel neurofeedback selectively modulates confidence without changing perceptual performance. *Nature Communications,* 7, 13669, doi:10.1038/ncomms13669.

Galvin, S. J., Podd, J. V., Drga, V., and Whitmore, J. (2003). Type 2 tasks in the theory of signal detectability: Discrimination between correct and incorrect decisions. *Psychon Bull Rev*., 10, 843-876.

Gold, J., Bennett, P. J., and Sekuler, A. B. (1999). Signal but not noise changes with perceptual learning. *Nature*, 402, 176-178.

Kiani, R., Corthell, L., and Shadlen, M. N. (2014). Choice certainty is informed by both evidence and decision time. *Neuron,* 84, 1329-1342, doi:10.1016/j.neuron.2014.12.015.

Kiani, R., and Shadlen, M. N. (2009). Representation of Confidence Associated with a Decision by Neurons in the Parietal Cortex. *Science,* 324, 759-764, doi:10.1126/science.1169405.

Macmillan, N. A., and Creelman, C. D. (2005). *Detetion theory: A user's guide*. 2nd edn, Cambridge, UK: Cambridge University Press.

Maniscalco, B., and Lau, H. (2012). A signal detection theoretic approach for estimating metacognitive sensitivity from confidence ratings. *Conscious Cogn*, 21(1), 422-30, doi:10.1016/j.concog.2011.09.021.

Pleskac, T. J., and Busemeyer, J. R. (2010). Two-stage dynamic signal detection: a theory of choice, decision time, and confidence. *Psychological review,* 117, 864-901, doi:10.1037/a0019737.

Ratcliff, R., and Starns, J. J. (2013). Modeling confidence judgments, response times, and multiple choices in decision making: recognition memory and motion discrimination. *Psychological review,* 120, 697-719, doi:10.1037/a0033152.
